# Recent Advances in the Determination of Milk Adulterants and Contaminants by Mid-Infrared Spectroscopy

**DOI:** 10.3390/foods12152917

**Published:** 2023-07-31

**Authors:** Carlotta Ceniti, Anna Antonella Spina, Cristian Piras, Francesca Oppedisano, Bruno Tilocca, Paola Roncada, Domenico Britti, Valeria Maria Morittu

**Affiliations:** 1 Department of Health Sciences, University of Catanzaro Magna Græcia, 88100 Catanzaro, Italy; ceniti@unicz.it (C.C.); aa.spina@unicz.it (A.A.S.); foppedisano@unicz.it (F.O.); tilocca@unicz.it (B.T.); roncada@unicz.it (P.R.); britti@unicz.it (D.B.); morittu@unicz.it (V.M.M.); 2 Interdepartmental Center Veterinary Service for Human and Animal Health, University of Catanzaro Magna Græcia, CISVetSUA, 88100 Catanzaro, Italy

**Keywords:** milk, food safety, food control, adulteration, traceability, infrared spectroscopy, mid-infrared, Fourier-transform infrared

## Abstract

The presence of chemical contaminants, toxins, or veterinary drugs in milk, as well as the adulteration of milk from different species, has driven the development of new tools to ensure safety and quality. Several analytical procedures have been proposed for the rapid screening of hazardous substances or the selective confirmation of the authenticity of milk. Mid-infrared spectroscopy and Fourier-transform infrared have been two of the most relevant technologies conventionally employed in the dairy industry. These fingerprint methodologies can be very powerful in determining the trait of raw material without knowing the identity of each constituent, and several aspects suggest their potential as a screening method to detect adulteration. This paper reviews the latest advances in applying mid-infrared spectroscopy for the detection and quantification of adulterants, milk dilution, the presence of pathogenic bacteria, veterinary drugs, and hazardous substances in milk.

## 1. Introduction

In recent decades, the consumption of milk has increased, and contextually, it is expected that world milk production will grow by 1.7% each year by 2028 [1]. Thanks to its constituents, such as proteins, fats, fatty acids, peptides, minerals, and vitamins, milk is considered to be a high nutritional value product and plays a relevant role in human nutrition [2,3,4].

The increased consumption and demand for milk production, along with the centrality of milk in human nutrition, have led to a situation where production is unable to meet the difference between supply and demand. This has resulted in milk becoming the target of numerous unethical procedures and has led to illegal adulteration practices to bridge this gap and increase profits, which could have severe effects on human health [5]. Moreover, milk payment is often based on multiple parameters such as bacteriological quality, protein, and fat percentage, making it susceptible to several potential milk adulterants [6].

Food adulteration and contamination events aiming to achieve economic gains or mask unsuitable conditions, including those seen in the milk industry, seem to occur with some regularity. For example, the contamination of milk with melamine, added with the purpose of increasing the nitrogen concentration and falsely inflating protein concentration, first emerged in China in 2008, causing worldwide concerns [7]. Moreover, globalization and rapid worldwide delivery systems could have far-reaching impacts. Thus, milk adulteration has become a serious problem in the food industry from both commercial and health perspectives [8]. An additional issue relates to the presence of veterinary drug residues in milk, which raises serious concern as these residues constitute potential threats to human health [9]. To protect consumers from further sources of antibiotics, maximum residue limits (MRLs) for veterinary drug residues in animal foodstuff raw materials have been set [10].

Food authentication, the process that verifies whether food complies with the description on the label, is a rapidly growing field. This growth is attributed, on one hand, to the increasing public awareness of food safety and, on the other hand, to the greater willingness of consumers to pay a higher price for products considered to be of high quality, such as those with Protected Designation of Origin [11]. Proof of provenance is an important topic for milk safety since these products are often the target of fraudulent labeling practices.

**Figure 1:**
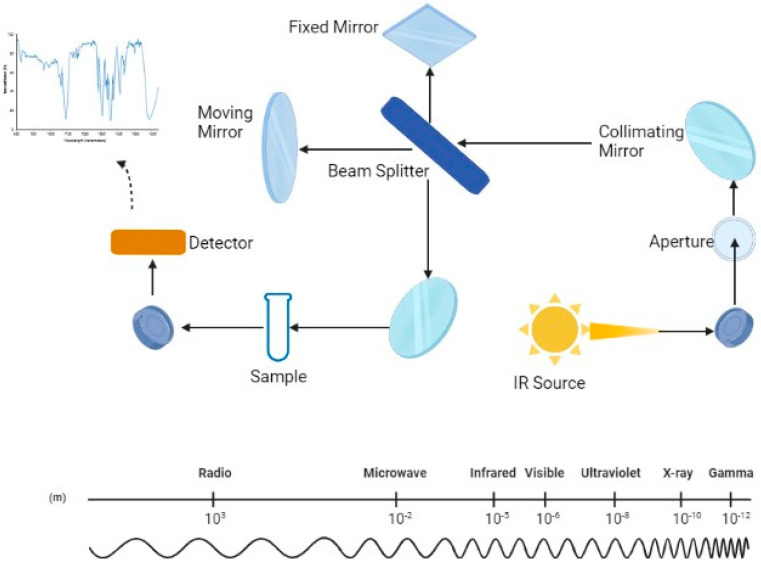
Schematic representation of the optical system of the mid-infrared spectrometer (MIRS) and, below, the wavelength scale; Created with Biorender.com.

In this scenario, the interest in analytical technologies, more rapid and cost effective than traditional tools, that can routinely and accurately measure the quality traits of milk has led to the application of infrared spectroscopy. Infrared spectroscopy is a technique based on the measurement of the wavelength and the intensity of absorption of infrared light by a substance [12]. In a few words, each functional group of a molecule has a unique vibrational frequency that can be used to determine what functional groups are present in a specific sample. Based on the wavelength, we distinguish different regions including the radio frequency (1 cm–1 m), the microwave region (100 µm–1 cm), the X-ray region (0.5–10 nm), the mid-infrared region (MIR) (2500–25,000 nm), the near-infrared region (NIR) (800–2500 nm), the visible region (350–800 nm), and the UV region (10–350 nm) [13] (Figure 1).

Mid-infrared spectroscopy (MIRS) is one of the most relevant technologies in the dairy industry for predicting the chemical and technological properties of different food matrices, and one of the most promising fields of application is milk [13,14,15,16,17]. In the mid-infrared region (2500 to 25,000 nm), electromagnetic radiation passes through matter, causing movements (e.g., vibration and rotation) of molecules through molecular bonds, resulting in varying degrees of energy absorption. By analyzing the energy supplied and the quantity absorbed by the sample, it is possible to determine the chemical composition of the examined sample [13]. Figure 1 summarizes the MIRS optical system and the wavelength scale through a schematic diagram.

The use of MIRS has become widespread, especially in milk, due to several advantageous features. This technology is non-destructive, does not require sample pretreatment, and the analysis of the sample does not require highly specialized personnel. Furthermore, spectra are obtained automatically, and they can be used simultaneously for multiple chemical and physical predictions. Handheld tools can also be used at the farm level for continuous monitoring “online”. The spectral data provide a detailed description of samples, acting as a unique “fingerprint” that reflects both physical states and molecular structures. These spectral data can be easily stored, and past records can be reevaluated when new calibrations become available [18,19]. Often, spectral data is pretreated and combined with chemometrics, which can enhance the reliability and reproducibility of the analysis results [13,20]. Chemometrics, defined as “the discipline that provides maximum information from chemical data,” appears to be highly suitable for the spectroscopy field [21]. Grassi and colleagues (2023) [22] presented an overview of different chemometric techniques for detecting milk adulteration, describing in detail the differences between discriminant modeling and classification methods.

Fourier-transform infrared (FTIR) spectrometry, a type of MIRS, facilitates the rapid scanning of a complete spectrum of electromagnetic waves, ranging from 4000 cm^−1^ to 400 cm^−1^ [19,23]. It is widely used to analyze milk samples and predict the content of fat, protein, lactose, and casein, which are collected periodically according to the milk-recording schemes of different countries. FTIR spectrometry has been authorized by The International Committee for Animal Recording (ICAR, 2012) as the standardized routine system for analyzing the constituents of milk.

Over the last few decades, Fourier-transform infrared spectrometry has also been proposed for the analysis of other milk components. The effectiveness of MIRS has been studied to predict phenotypes for dairy industry applications, such as coagulation properties, fatty acid and protein composition, acidity, mineral composition, body energy status, ketone bodies, and methane emissions, as extensively reviewed by DeMarchi [13]. It has also been utilized for quality evaluation of milk products [24]; more recently, in response to fraudulent practices and health concerns regarding milk, MIRS, often supported by chemometric approaches, has also been employed to detect milk authenticity and adulteration, proving to be a promising tool compared to traditional techniques [25,26]. Nascimento [27] reviewed the literature on the main milk adulterants, analytical techniques, and sample treatment strategies from 2010 to 2016, highlighting that milk adulteration is a topic of general concern.

There is an insistent and growing need in the food industry to develop simpler, quicker, more cost-effective, and more efficient methods for detecting chemical substances, adulterants, and veterinary drugs in milk, and to develop accurate predictive models for quantifying the addition of these harmful substances that affect the quality of milk. Therefore, in this review, we aim to provide a reprisal of the most relevant literature on mid-infrared spectroscopy studies performed over the last two decades. Literature searches and reviews have been conducted, taking into account the recent applicative potential of MIRS in milk. Special attention is devoted to main milk adulterants, milk contaminants, and residues of veterinary drugs, within the context of food safety. Additionally, this work emphasizes the potential role of MIRS in the detection and monitoring of microbial pathogens potentially present in milk.

## 2. Adulterants, Diluents, Chemical Substances, and Mycotoxins in Milk

When we define food adulteration, we refer to the addition or subtraction of any substance that affects the quality of the food. Milk adulteration has become a severe global threat, posing risks to consumer health and causing economic losses in the dairy sector. Consequently, there is a growing interest in techniques that can accurately and consistently detect adulterants and chemical substances. Some authors have highlighted the use of FT-MIR as a rapid method for detecting food adulterants or quality defects in fresh milk or milk powder.

The primary milk adulterations involve adding water and using milk from other undeclared milk species. Adding water is a common practice to increase the volume of milk, but it can be easily detected by measuring the depression of the freezing point [28]. However, this method may not always be effective, as adulterants can mask its effects. Total reflection MIRS and partial least squares discriminant analysis (PLS-DA) have been applied to detect and quantify water and other contaminants in milk [29]. Though the method was able to detect five contaminants, including water, accurately quantifying the adulteration with water proved challenging (with false positive and false negative rates of 4% and 3.2% in the test set and 11.5% in the training sets) due to the substantial water content naturally present in milk and physiological variations.

A similar conclusion was reached by Gondim and colleagues [30]. In fact, in the case of water, the specificity of the one-class model (unadulterated milk samples) was lower than 70% (56.7), which means that the samples of milk adulterated with water cannot be effectively differentiated from unadulterated samples.

Adding water to milk results in a decrease in nutritional substances, such as protein and solid content [31]. To counter the dilution of milk caused by adding water, adulterants such as sucrose are often used to increase the total solid content and sweeten the milk. FTIR spectroscopy, in combination with multivariate analysis, has been successfully applied to discriminate between sucrose-adulterated milk and pure milk. The spectral regions 3800–2800 cm^−1^ and 1800–900 cm^−1^ and two key prominent peaks were observed in the adulterated milk samples (996 cm^−1^ and 1052 cm^−1^). These characteristic peaks identified in adulterated milk samples were not present in pure milk and likely correspond to the glycosidic linkage in sucrose [32].

Adding water to milk results in a decrease in nutritional substances, such as protein and solid content. To address this, cheaper nitrogen-rich compounds and urea are sometimes added to simulate higher milk protein content [33]. Attenuated total reflectance-Fourier-transform infrared (ATR-FTIR) spectroscopy has been used to detect and quantify added urea in milk [34]. The authors identified clear differences among milk spectra with and without urea supplementation, in the region 1670–1564. Adulteration of milk with substances that mask the dilution of milk not only results in major economic losses for the food processing industry but also exposes consumers to health risks. This concept can be well represented by the case of melamine addition.

Melamine (2,4,6-tri amino-1,3,5-triazine), usually employed as an industrial chemical compound in the production of formaldehyde resins, is not approved as an ingredient or additive in food, but some producers illegally used it as an adulterant to augment the protein content. The unscrupulous practice of mixing chemical compounds, including melamine, with milk to boost the protein content is widely known. In China and other countries, the case of melamine-contaminated food, including raw milk [35], first emerged in 2008 [7,36].

The methodology based on “fingerprint” has proven to be a strategy that can confirm the presence of the analyte of interest. Mauer and colleagues applied FTIR methods [37] to aid the rapid detection of 1 ppm melamine in infant formula powder. Balabin [38] proposes the use of spectroscopy data produced by MIRS coupled for melamine detection in dairy matrixes, including liquid milk, showing that it is an effective tool to detect melamine with a limit of detection below 1 ppm (0.76 ± 0.11 ppm). Jawaid [39] collected spectra of milk samples in the mid-infrared region (4000–650 cm^−1^) to quantify and detect melamine in raw and powder milk samples; the author applied partial least squares (PLS) models to correlate milk spectral data to melamine concentration (R2 > 0.99, and RMSEC 0.370), underling how this tool provides information on the structure of organic compounds and thus proposing this method for fast analysis. Attenuated total reflectance-Fourier-transform infrared (ATR-FTIR) in conjunction with single-class soft independent modeling of class analogy (SIMCA) model was applied to detect melamine in milk powder **[40]**. The author obtained a satisfactory prediction with a correct classification rate of 100% for test samples ≥ 0.30% mixed wet and 1.0% mixed dry using spectra in the range 850–750 cm^−1^. Although the commonly used approach to detect melamine, including liquid chromatography or gas chromatography, is accurate (0.3 ppm) [41], the advantages of these MID-IR techniques lie in simplifying the analytical process.

Even milk fats could be subject to fraudulent operations such as their replacement with non-dairy, cheaper oils or fats that will result in a lower saponification value, the most common quality index [42]. It is very complex to identify this type of adulteration as very often the addition of foreign fat does not induce a change in flavor, and the saponification value is not a certain indicator in detecting fraud if the adulterant is coconut oil, whose saponification value is higher [42]. In a very interesting work, Hanganu and Chira [43] highlighted the weaknesses of 1H-NMR spectroscopy for the detection of milk and dairy products adulterations, demonstrating that the butyric acid may be confounded with the linolenic acid due to the overlapping of their distinctive signals at 0.96 ppm, thus leading to confusion and false classification results. Also, in this case, the Fourier-transform coupled with chemometrics proved to be a valid tool for detecting the adulteration of ghee with coconut oil at different concentrations (2, 4, 6, 8, 10, and 15%); analyzing the wavenumber region of 4000–500 cm^−1^ of 240 samples, the principal component analysis (PCA) showed the distinct clustering of pure ghee samples and coconut oil adulterated samples (till 2% adulteration) based on the selected wavenumber range (1170–1141 and 1117–1100 cm^−1^) [44].

Some authors applied MIR in the determination of a group of adulterants. Da Costa Filho and colleagues applied FTIR-MIR as a rapidly screening for detecting sixteen adulterants in reconstituted skimmed milk powder, proposing this tool to rapidly screen raw materials and detect abnormalities [45]. The author reported that the method correctly identified 100% of unadulterated samples and 93% of samples adulterated with nitrogen-rich compounds, thus showing the presence of characteristic peaks of food adulterants at 5% economic adulteration. In another interesting work, Neto [46] applied machine learning techniques to milk spectral data. The milk samples were adulterated with five different substances, namely, sucrose, soluble starch (amylose and amylopectin), sodium bicarbonate, hydrogen peroxide, and formaldehyde, with promising results, showing classification accuracies up to 98.76%. Conceição applied FTIR and multivariate analysis identifying (from 0.1%) milk adulterated with a set of six adulterants: urea, sodium hydroxide, sodium bicarbonate, hydrogen peroxide, starch, and sucrose [47]. Hansen and colleagues [25] focused their work on describing how targeted and untargeted models applied to FT-IR spectroscopy with commercial equipment for routine milk analysis could detect potential milk adulterants, giving a practical point of view underlying the advantage or disadvantages of these two models.

Another relevant topic is the accidental or intentional presence of lactose in low-lactose products. The demand for low-lactose products, including milk, has become widespread due to the high number of consumers with low lactase enzyme production and, thus, with lactose intolerance. Ribeiro and colleagues [48] applied the association of FTIR with machine learning tools as an original proposal to detect and quantify residual lactose and other sugars in low-lactose milk. The results of this interesting study performed on raw milk, pasteurized milk, and ultra-high temperature (UHT) milk indicated good accuracy (95%) for classification. Moreover, the coefficient of determination (R2) was 81%, 86%, and 92%, respectively, for lactose, glucose, and galactose quantification, suggesting how this approach could be useful to identify and quantify sugars in low-lactose milk in a fast execution time.

Another interesting topic is the contamination of milk with aflatoxins, which pose a serious health hazard to consumers as they are potentially carcinogenic compounds [49]. The results obtained by Jaiswal and colleagues [50] revealed significant differences among pure and AFM1 spiked milk samples (0, 0.02, 0.04, 0.06, 0.08, and, 0.1 μg/L) spectra in the regions of 1800–650 cm^−1^ and 3689–3499 cm^−1^, probably attributed to the chemical structure of AFM1. The model built with principal component analysis (PCA) showed significant clustering of bovine milk samples (*p* < 0.05) and could classify more than 86% of milk samples contaminated even with a low concentration of AFM1 (0.02 μg/L). The analysis and identification of aflatoxins in milk could be more challenging than other adulterants due to the low presence of the molecule; furthermore, the literature to date is limited on this point and deserves further study.

## 3. Milk Speciation and Geographical Origin

Currently, agricultural product trademarks, such as Protected Designation of Origin (PDO), Geographical Indication Products (PGI), and Traditional Specialty Guaranteed (TSG), are gaining attention from consumers who prefer them over unbranded products [51]. However, preserving these productions requires initiatives to protect against fraud, which commonly includes the substitution of one component for a cheaper similar one and the sale of products falsely marketed as “local” or from specific geographic regions [52].

In 2015, AOAC International (Association of Official Analytical Chemists) approved and recommended the FTIR method for determining fat, protein, lactose, and casein in milk and dairy products [53]. Over the past decade, FTIR spectroscopy, combined with chemometric techniques, has shown potential for detecting authenticity and adulteration in milk samples. This aspect is important since milk is the second most at-risk food item for adulteration, after olive oil [54], and researchers have explored this approach for milk authentication.

Encouraging results have been achieved in studies on milk adulteration with species other than those declared. For instance, Spina and colleagues [55] explored the potential application of mid-infrared (MIR) spectroscopy in combination with PLS regression analysis to detect adulterations in buffalo milk with cow milk. Buffalo milk, which is more expensive than cow milk, is widely used in Italy for mozzarella cheese production, holding the European “Protected Designation of Origin” (PDO) status to prevent fraud. The authors successfully used FTIR technology to detect a small portion of 3% cow milk in buffalo milk, reaching good accuracy.

In another study, results on 165 buffalo milk samples adulterated with cow’s milk (10 to 90%) were analyzed using mid-infrared spectroscopy combined with PLS and PCA, enabling classification of adulteration with a calibration error of about 5% [56].

The FTIR spectrophotometer measurement and PLS calibration also brought good results with adulterated camel milk samples. Souhassou and colleagues showed low relative error (3.8%), and the method was proposed to be a valid method for the authentication of camel milk from cow milk [57].

FTIR spectroscopy and PLS regression also provided reliable predictions for the adulteration of goat milk with cow milk above 5% level (*v*/*v*) [58]. In an interesting study, Pappas and colleagues (2008) [59], through FT-IR spectroscopy and discriminant analysis (cluster analysis), accurately distinguished all sheep and goat milk samples, showing that goat milk samples can be differentiated from sheep milk samples. Nicolau et al. (2010) [60] demonstrated that FT-IR spectroscopy in combination with PLS or with Kernel Nonlinear PLS (KPLS) is an easy to perform, accurate and rapid (30 s for samples) method for the quantitative assessment of sheep, goat, and cow milk in binary and tertiary mixtures. The authors found an error (5–8%) for all the species observed and mixed samples, reaching better predictive results (4–6%) when KPLS was employed.

The chemical and nutritional composition of the milk is influenced by several factors; genetics represents a crucial point, not only as in terms of the species but also the breeds of dairy animals [61]. In addition, some dairy products from various countries around the world may be produced only from the milk of specific breeds to guarantee nutritional and sensory characteristics. In this context, it appears more difficult to distinguish through using MID technology the adulteration of milk samples with milk belonging to the same species but different breeds.

As reported by Sallhe [61], Fourier-transform infrared spectroscopy (FTIR) coupled with multivariate analysis (PLS) showed predictive features in differentiating milk from different goat breeds. In this investigation, 18 goat milk samples belonging to Jamnapari, Saanen, and Toggenburg breeds were analyzed, and the results showed that the model classifies the goat milk according to the breeds, using functional group profiles as predictors of model validity and predictivity (Q^2^ and R^2^Y values were 0.981 and 0.958).

Animal species and breed alone cannot determine the final quality of the product and their commercial value. The geographical origin could confer organoleptic qualities associated with regional products. Thus, the development of new procedures for determining the geographical origin of milk and dairy products is highly useful and desirable. Various studies have employed NIR and MID technology to successfully identify the exact geographical origin of dairy products [51,62,63,64]; However, there are still a few works that trace the geographical area of milk through MID technology. In Italy, predictive models of the geographical origin of sheep milk were built on fatty acid composition using MIR spectra. All models correctly predicted (correct predictions of 96%) the geographical origin of 250 samples of sheep milk coming from different areas of the region of Sardinia [65].

**Figure 2:**
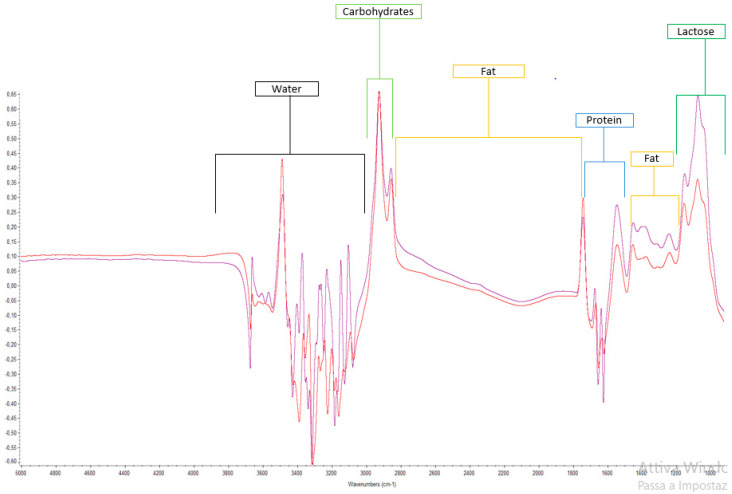
Representative images of the mid-infrared spectrum of milk and the approximate putative region obtained with TQ Analyst software ver. 8.0 (Thermo Fisher Scientific, Madison, WI, USA).

Scampicchio and Colleagues (2016) [66] explored the potentiality of a routine chemical analysis tool (Milkoscan^®^) combined with a chemometric model (PLS-DA) to identify and discriminate the geographical origin of milk samples coming from Alpine (North and South Tyrol area) and milk samples coming from other European regions. In this study, the cow milk samples were discriminated according to their geographical origin with an error lower than 5%, taking into account fatty acids content, dry matter, and freezing point as the most important contributors to the variance. Table 1 summarizes the applications of mid-infrared (MIR) spectroscopy to the analysis of milk mixture samples. Generally speaking, the main bands obtained in the spectra, reported in the literature, were connected to the presence of functional groups belonging to water, lipids, and proteins in the range between 4000 and 1500 cm^−1^ [24]. The major peaks in the spectra range of 1500–1700 cm^−1^ are proteins. In protein spectra, the amide bands originate from the vibration of peptide groups, and in particular, absorption in this area has been assigned to amide I and amide II [67]. An absorption peak at approximately 1400 cm^−1^ and 1700 cm^−1^ is attributed to lipids and fats. The band in the 2800–3000 region represents carbohydrates, while the 900–1200 cm^−1^ region is associated with lactose. For fatty acids, the selected regions for most fatty acid categories are included approximately in the spectral subsets 950–900 cm^−1^, 1000–1250 cm^−1^, 1700–1800 cm^−1^, and 2800–2900 cm^−1^ [65]. Finally, peaks between 3400 and 3000 cm^−1^ and between 1700 and 1500 cm^−1^ correspond to the O-H stretching and O-H folding regions, respectively, thus indicating the presence of water in these regions of the spectrum [30]. Figure 2 summarizes these putative regions.

The potential of using fluorescence in food research has increased in recent years, and future research should focus on specific calibrations or markers for milk species or geographical origin.

## 4. Pathogens, Biofilm, and Microbial Toxins

Another great advantage of MIR spectroscopy lies in its capability to elucidate biological macromolecules as the fingerprint of microorganisms commonly associated with reduced food quality, spoilage, or pathogenic potential. For instance, Jiang and colleagues [69] studied the surface composition of Gram-positive and Gram-negative bacteria and their isolated cell walls via attenuated total reflectance Fourier-transform infrared (ATR-FTIR) spectroscopy in the mid-infrared (4000 to 400 cm^−1^) spectral regions. Recent advances in detector technology and data manipulation such as multivariate analysis enabled the thorough detection of microbial specimens in a variety of food matrixes. Studies from McKnight employed FTIR spectroscopy for the efficient detection and discrimination of a plurality of pathogenic specimens such as Escherichia coli O157:H7, Pseudomonas aeruginosa, Salmonella enterica, Bacillus cereus, Enterobacter sakazakii, Listeria spp., and Alicyclobacillus in fruit juices and drinking water [70]. A detailed list of the milestone studies employing MIR along with multivariate data analysis to detect and discriminate pathoagenic and/or food detrimental bacterial specimen is provided in some articles [71,72]. As an example, by using FT-IR in combination with PLSR, Nicolaou and Goodacre [73] rapidly acquired milk fingerprints and quantified the microbial amount in milk samples. The authors concluded that FT-IR has great potential in the dairy industry as a screening method for detection and enumeration, with very little sample preparation. However, in non-milk matrices, Moreirinha et al. [74] successfully employed mid-infrared spectroscopy to confirm the presence of Listeria spp. and Salmonella spp. isolated from cheeses, sausages, and prepared dishes, underlining the potential of this technology as a sensitive and rapid alternative to detect foodborne pathogens of One Health relevance.

Besides the detection of planktonic cells, MIR holds the potential of measuring the biofilm typically associated with food matrices. In the food industry, biofilms represent an important threat to food safety and quality since microorganisms present in biofilms can remain in food processing, increasing the chances of food contamination and foodborne infections. In addition, strong resistance to antimicrobial agents is being registered as compared to the planktonic counterparts, raising the need for new strategies for the early and rapid detection of biofilm across the food processing stages. In this perspective, a valid alternative is given by mid-infrared spectroscopy, the usage of which enabled the characterization of the macromolecular composition’s biofilm matrices and the assessment of the suitability of this method for assessing the dynamics of onset and evolution of heterogeneous biofilms, including fungi, algae, and protozoa [71,75]. Bosch et al. [76] characterized the biofilm of B. pertussis, describing higher intensity in the absorption bands relative to the polysaccharides (1200–900 cm^−1^) and carboxylate groups (1627, 1405, and 1373 cm^−1^) when compared with the spectra of the same bacterial cells growing in the planktonic form. More recently, Wang and colleagues [77] explored mid-infrared spectroscopy intending to detect changes in the composition of spoiled milk due to the different thermophilic bacteria biofilm forming, showing that within the range of 3000–2800 cm^−1^, the control group and the putrid milk groups were significantly separated. However, two limitations of this technique are highlighted: (i) only the base layer of biofilms provides biochemical information; (ii) a higher spectral absorption noise is registered, so an efficient calibration of the instrument is necessary [71]. Nevertheless, this technique offers the advantages of being simple, fast, easy to use, non-destructive, and environmentally friendly, making it a promising approach for routine analyses and potentially implemented in official food control as a screening strategy complementing gold standard approaches.

## 5. Drug Residues

The presence of antibiotic residues and other contaminants in milk represents a great challenge for public health. In case their concentration exceeds the established maximum limits, those molecules may negatively impact human health. Developing a rapid, high-throughput, and economically convenient method is required to detect these kinds of molecules. From this perspective, infrared spectroscopy represents a platform useful for this purpose. The detection of antibiotics or antibiotic residues in milk is feasible with immunochemical methods [78] or with mass spectrometry [79]. However, both methods have some flaws. The first one lacks multiplexing, and the second one is expensive and requires laborious sample preparation protocols and trained personnel to guarantee data reliability. On the other hand, infrared spectroscopy applied to milk analysis is high throughput, cost effective, and offers multiplexing features. Moreover, it is already widely used for the routine analysis of milk of several species for human consumption. Moreover, as described below, it has been already used for the detection of antibiotics and proteins such as lactoferrin, placing this versatile method as a good choice for high-throughput routine analysis.

In 2018, Casarrubias-Torres et al. conducted a study coupling Mid-FTIR (Fourier-transform mid-infrared (FTIR) spectroscopy) with chemometric analysis in order to detect and quantify the presence of three different tetracycline antibiotics in cow’s milk. In particular, the applied method facilitated the rapid detection of these antibiotics at concentrations of μg/L. The study was conducted on 30 cow’s milk samples to which tetracycline, chlortetracycline, and oxytetracycline were added in a concentration of 10–400 μg/L. The analyzed samples showed different spectra, as a higher content of antibiotics determines a higher absorbance. Therefore, for each sample a variation in the absorption of infrared energy was recorded due to the different presence of the functional groups, thus reporting changes in the Mid-FTIR region. Subsequently, chemometric analysis using the soft independent modeling of class analogy (SIMCA) model made it possible to discriminate between milk, milk added with tetracycline, milk added with chlortetracycline, and milk added with oxytetracycline. Therefore, Mid-FTIR and chemometric analysis made it possible to rapidly detect and quantify the antibiotics present in cow’s milk at low concentrations, i.e., with a limit of detection (LOD) > 10 μg/L. This value is considered acceptable as it is in accordance with the maximum residue limits (MRLs) of veterinary drugs present in human food and established in 300 μg·L^−1^ by the US Food and Drug Administration (FDA) and in 100 μg·Kg^−1^ by the European Union, for tetracycline residues present in bovine milk [80]. The potential of Mid-FTIR, associated with chemometric, to rapidly detect and quantify the presence of tetracycline hydrochloride residues in milk, even at low concentrations, had already been demonstrated by Sivakesava et al. in 2002. The study reported optimal correlation coefficients between 0.90 and 0.92 [81]. Furthermore, the FTIR spectra (in particular, attenuated total reflection Fourier-transform infrared (ATR-FTIR) spectroscopy) associated with multilayer perceptron network (MLP) and partial least squares (PLS) allowed us to identify and quantify the presence of tylosin residues directly in the fluid milk. Tylosin is a macrolide antibiotic often used in the dairy industry, and its MRL is 50 µg/kg. FTIR with MLP and PLS detected tylosin residues in the range of 0–100 µg/mL with a high correlation (R ≥ 0.99), confirming the efficiency of the method for food safety [82]. In addition, in 2021, de Freitas et al. published a study in which they determined the presence of tylosin residues, in low concentrations (≤100 μg·L^−1^), in powdered milk, using FTIR spectroscopy in association with random forest [83]. In particular, the milk used was obtained from healthy Holstein/Zebu cows, not treated with antibiotics in the 2 months preceding the study. The liquid milk was divided into aliquots, some of which were adulterated by the addition of tylosin from 10 to 100 μg·L^−1^. Subsequently, all the aliquots were lyophilized before being subjected to spectroscopic analysis. This methodology was also successfully applied to directly analyze powdered milk, quickly and efficiently. Furthermore, Teixeira et al. conducted a theoretical, experimental, and chemometric study to determine the presence of β-lactam antibiotics, such as penicillin and ampicillin, in low concentrations in cow’s milk. The infrared and Raman spectra together with the density functional theory (DFT) and the statistical analysis principal component analysis (PCA) allowed us to discriminate the behavior of penicillin and ampicillin in water and milk [84]. Table 2 summarizes the literature evidence of antibiotics detection through FTIR spectroscopy.

Mid-infrared spectrometry was also utilized by Soyeurt et al. to establish an equation capable of quantifying the lactoferrin (LTF) content in bovine milk. LTF is a glycoprotein naturally present in milk, and its concentration increases during lactation. However, it also serves as a marker of inflammation and acts as a general antibacterial and antifungal molecule. Determining its content could aid in identifying mastitis in dairy cows when combined with somatic cell score (SCS).

The study was conducted using milk samples from Belgium, Ireland, and Scotland, obtained from cows of various breeds and different production systems. Based on the mid-infrared spectra, the LTF equation was derived with a cross-validation coefficient of determination of 0.71 and a cross-validation standard error of 50.55 mg/L of milk. This research demonstrates that mid-infrared spectrometry facilitates the rapid quantification of LTF directly in bovine milk, enhancing the identification of clinical mastitis when used in conjunction with SCS alone. Moreover, it provides an opportunity to improve the nutritional quality of milk [85].

## 6. Final Remarks and Future Outlook

Over the last two decades, the application of mid-infrared (MID)-spectroscopic techniques for the analysis of milk adulterants has significantly grown. Using official methods as screening tools for numerous milk samples and various types of adulterants is neither practical nor cost effective. The food industry has shown interest in new analytical technologies that are faster and less expensive than traditional methods, and MID-IR spectroscopy offers several advantages, such as ease of use, non-destructive sample preparation, and rapid identification of multiple parameters. As a result, it can be applied practically to detect milk adulteration, making it a promising alternative or complementary method to existing food safety detection methods. This technique holds potential for identifying species and geographical origin as well. Although the literature on the identification of pathogenic microorganisms in milk is not yet consistent, Fourier-transform infrared spectroscopy (FT-IR) could be a versatile technique for the rapid differentiation, classification, identification, and screening of microorganisms. However, further research is necessary to fully explore the potential of MIR spectroscopy in detecting possible hazardous substances in milk.

## Figures and Tables

**Table 1:** Applications of mid-infrared (MIR) spectroscopy to the analysis of milk mixture samples.

Samples	Aim	Methods	Regions (Wavenumber Range cm^−1^)	Accuracy	References
Buffalo milk	Determination and quantification of cow milk in buffalo milk	FTIR + PLS	1000–3000	R2 = 0.99	[55]
Buffalo milk	Determination and quantification of cow milk in buffalo milk	MID + PCA, PLS.	1000–3000	R2 = 0.98	[68]
Buffalo milk	Determination and quantification of cow milk in buffalo milk	FTIR + OPLS-DA	650–4000	R2 = 0.98	[58]
Sheep milk	Determination and quantification of cow milk in sheep milk	FTIR + Kernel PLS	600–4000	R2 = 0.95	[60]
Goat milk	Determination and quantification of cow milk in goat milk	FTIR + Kernel PLS	600–4000	R2 = 0.84	[60]
Camel milk	Determination and quantification of cow milk in camel milk	FTIR + PLS	920–3600	R2 = 0.99	[57]
Goat Milk	Determination of milk samples to different goat breeds	FTIR + PLS-DA	950–3000	R2 = 0.95	[61]
Sheep Milk	Determination of milk samples to different geographical origin	FTIR + LDA; PCA	926–3500	R2 = 0.99	[65]
Cow milk	Determination of milk samples to different geographical origin	FTIR + PLS-DA	1000–4000	R2 = 0.93	[66]

**Table 2:** Literature evidence of antibiotics detection through FTIR spectroscopy.

Antibiotic	Limit of Detection (LOD)/Range	Accuracy	References
Tetracycline	LOD = 10 µg/L	R2 = 0.99	[80]
Chlortetracycline	LOD = 10 µg/L	R2 = 0.99	[80]
Oxytetracycline	LOD = 10 µg/L	R2 = 0.99	[80]
Tetracycline	Range 4–2000 ppb	R2 = 0.89	[81]
Tylosin	Range 0–100 µg/L	99%	[82]
Tylosin (powdered milk)	Range 0–100 µg/L	R2 ≥ 0.95	[82]

## Data Availability

Not applicable.

## References

[1] (2019). CD-FAO Agricultural Outlook 2019–2028.

[2] Stergiadis S., Berlitz C.B., Hunt B., Garg S., Givens D.I., Kliem K.E. (2018). An update to the fatty acid profiles of bovine retail milk in the United Kingdom: Implications for nutrition in different age and gender groups. Food Chem..

[3] Miller G., Jarvis J., McBean L. (1999). Handbook of Dairy Foods and Nutrition.

[4] Muehlhoff E., Bennett A. (2013). Milk and Dairy Products in Human Nutrition—Question and Answers.

[5] Handford C.E., Campbell K., Elliott C.T. (2015). Impacts of Milk Fraud on Food Safety and Nutrition with Special Emphasis on Developing Countries. Compr. Rev. Food Sci. Food Saf..

[6] Das S., Goswami B., Biswas K. (2016). Milk Adulteration and Detection: A Review. Sens. Lett..

[7] Chan E., Griffiths S., Chan C. (2008). Public-health risks of melamine in milk products. Lancet.

[8] Ellis D.I., Brewster V.L., Dunn W.B., Allwood J.W., Golovanov A.P., Goodacre R. (2012). Fingerprinting food: Current technologies for the detection of food adulteration and contamination. Chem. Soc. Rev..

[9] Sachi S., Ferdous J., Sikder M., Hussani S. (2019). Antibiotic residues in milk: Past, present, and future. J. Adv. Veter. Anim. Res..

[10] The European Commission (2010). Commission Regulation (EU) No 37/2010 of 22 December 2009 on pharmacologically active substances and their classification regarding maximum residue limits in foodstuffs of animal origin. Off. J. Eur. Union.

[11] Spink J., Moyer D.C. (2011). Defining the Public Health Threat of Food Fraud. J. Food Sci..

[12] Putzig C.L., Leugers M.A., McKelvy M.L., Mitchell G.E., Nyquist R.A., Papenfuss R.R., Yurga L. (1994). Infrared Spectroscopy. Anal. Chem..

[13] De Marchi M., Toffanin V., Cassandro M., Penasa M. (2014). Invited review: Mid-infrared spectroscopy as phenotyping tool for milk traits. J. Dairy Sci..

[14] Xiao S., Wang Q., Li C., Liu W., Zhang J., Fan Y., Su J., Wang H., Luo X., Zhang S. (2021). Rapid identification of A1 and A2 milk based on the combination of mid-infrared spectroscopy and chemometrics. Food Control.

[15] Manuelian C., Visentin G., Boselli C., Giangolini G., Cassandro M., De Marchi M. (2017). Short communication: Prediction of milk coagulation and acidity traits in Mediterranean buffalo milk using Fourier-transform mid-infrared spectroscopy. J. Dairy Sci..

[16] De Marchi M., Toffanin V., Cassandro M., Penasa M. (2013). Prediction of coagulating and noncoagulating milk samples using mid-infrared spectroscopy. J. Dairy Sci..

[17] Zhao X., Song Y., Zhang Y., Cai G., Xue G., Liu Y., Chen K., Zhang F., Wang K., Zhang M. (2023). Predictions of Milk Fatty Acid Contents by Mid-Infrared Spectroscopy in Chinese Holstein Cows. Molecules.

[18] Bittante G., Cecchinato A. (2013). Genetic analysis of the Fourier-transform infrared spectra of bovine milk with emphasis on individual wavelengths related to specific chemical bonds. J. Dairy Sci..

[19] Soyeurt H. (2023). Fourier transform mid-infrared milk screening to improve milk production and processing. JDS Commun..

[20] De Marchi M., Penasa M., Cecchinato A., Mele M., Secchiari P., Bittante G. (2011). Effectiveness of mid-infrared spectroscopy to predict fatty acid composition of Brown Swiss bovine milk. Animal.

[21] Aleixandre-Tudo J., Castello-Cogollos L., Aleixandre J., Aleixandre-Benavent R. (2022). Chemometrics in food science and technology: A bibliometric study. Chemom. Intell. Lab. Syst..

[22] Grassi S., Tarapoulouzi M., D’alessandro A., Agriopoulou S., Strani L., Varzakas T. (2022). How Chemometrics Can Fight Milk Adulteration. Foods.

[23] Karoui R., Downey G., Blecker C. (2010). Mid-Infrared Spectroscopy Coupled with Chemometrics: A Tool for the Analysis of Intact Food Systems and the Exploration of Their Molecular Structure−Quality Relationships—A Review. Chem. Rev..

[24] Anjos V. (2020). Federal University of Juiz de Fora Near and Mid Infrared Spectroscopy to Assess Milk Products Quality: A Review of Recent Applications. J. Dairy Res. Technol..

[25] Hansen P.W., Holroyd S.E. (2019). Development and application of Fourier transform infrared spectroscopy for detection of milk adulteration in practice. Int. J. Dairy Technol..

[26] Kamal M., Karoui R. (2015). Analytical methods coupled with chemometric tools for determining the authenticity and detecting the adulteration of dairy products: A review. Trends Food Sci. Technol..

[27] Nascimento C.F., Santos P.M., Pereira-Filho E.R., Rocha F.R. (2017). Recent advances on determination of milk adulterants. Food Chem..

[28] Barham G.S. (2014). Detection and Extent of Extraneous Water and Adulteration in Milk Consumed at Hyderabad, Pakistan. J. Food Nutr. Sci..

[29] Botelho B.G., Reis N., Oliveira L.S., Sena M.M. (2015). Development and analytical validation of a screening method for simultaneous detection of five adulterants in raw milk using mid-infrared spectroscopy and PLS-DA. Food Chem..

[30] Gondim C.S., Junqueira R.G., de Souza S.V.C., Ruisánchez I., Callao M. (2017). Detection of several common adulterants in raw milk by MID-infrared spectroscopy and one-class and multi-class multivariate strategies. Food Chem..

[31] Azad T., Ahmed S. (2016). Common milk adulteration and their detection techniques. Int. J. Food Contam..

[32] Balan B., Dhaulaniya A.S., Jamwal R., Yadav A., Kelly S., Cannavan A., Singh D.K. (2020). Rapid detection and quantification of sucrose adulteration in cow milk using Attenuated total reflectance-Fourier transform infrared spectroscopy coupled with multivariate analysis. Spectrochim. Acta Part A Mol. Biomol. Spectrosc..

[33] Santos P., Pereira-Filho E., Rodriguez-Saona L. (2013). Rapid detection and quantification of milk adulteration using infrared microspectroscopy and chemometrics analysis. Food Chem..

[34] Jha S.N., Jaiswal P., Borah A., Gautam A.K., Srivastava N. (2014). Detection and Quantification of Urea in Milk Using Attenuated Total Reflectance-Fourier Transform Infrared Spectroscopy. Food Bioprocess Technol..

[35] Yan N., Zhou L., Zhu Z., Chen X. (2009). Determination of Melamine in Dairy Products, Fish Feed, and Fish by Capillary Zone Electrophoresis with Diode Array Detection. J. Agric. Food Chem..

[36] Rey M., Enjalbert F., Combes S., Cauquil L., Bouchez O., Monteils V. (2013). Establishment of ruminal bacterial community in dairy calves from birth to weaning is sequential. J. Appl. Microbiol..

[37] Mauer L.J., Chernyshova A.A., Hiatt A., Deering A., Davis R. (2009). Melamine Detection in Infant Formula Powder Using Near- and Mid-Infrared Spectroscopy. J. Agric. Food Chem..

[38] Balabin R.M., Smirnov S.V. (2011). Melamine detection by mid- and near-infrared (MIR/NIR) spectroscopy: A quick and sensitive method for dairy products analysis including liquid milk, infant formula, and milk powder. Talanta.

[39] Jawaid S., Talpur F.N., Sherazi S., Nizamani S.M., Khaskheli A.A. (2013). Rapid detection of melamine adulteration in dairy milk by SB-ATR–Fourier transform infrared spectroscopy. Food Chem..

[40] Limm W., Karunathilaka S.R., Yakes B.J., Mossoba M.M. (2018). A portable mid-infrared spectrometer and a non-targeted chemometric approach for the rapid screening of economically motivated adulteration of milk powder. Int. Dairy J..

[41] Li J., Qi H.-Y., Shi Y.-P. (2009). Determination of melamine residues in milk products by zirconia hollow fiber sorptive microextraction and gas chromatography–mass spectrometry. J. Chromatogr. A.

[42] Ivanova M., Hanganu A., Dumitriu R., Tociu M., Ivanov G., Stavarache C., Popescu L., Ghendov-Mosanu A., Sturza R., Deleanu C. (2022). Saponification Value of Fats and Oils as Determined from ^1^H-NMR Data: The Case of Dairy Fats. Foods.

[43] Hanganu A., Chira N.-A. (2021). When detection of dairy food fraud fails: An alternative approach through proton nuclear magnetic resonance spectroscopy. J. Dairy Sci..

[44] Gandhi K., Sharma R., Seth R., Mann B. (2021). Detection of coconut oil in ghee using ATR-FTIR and chemometrics. Appl. Food Res..

[45] Filho P.A.D.C., Chen Y., Cavin C., Galluzzo R. (2022). Mid-infrared spectroscopy: Screening method for analysis of food adulterants in reconstituted skimmed milk powder. Food Control.

[46] Neto H.A., Tavares W.L., Ribeiro D.C., Alves R.C., Fonseca L.M., Campos S.V. (2019). On the utilization of deep and ensemble learning to detect milk adulteration. BioData Min..

[47] Conceição D., Gonçalves B.-H., Da Hora F., Faleiro A., Santos L., Ferrão S. (2018). Use of FTIR-ATR Spectroscopy Combined with Multivariate Analysis as a Screening Tool to Identify Adulterants in Raw Milk. J. Braz. Chem. Soc..

[48] Ribeiro D.C., Neto H.A., Lima J.S., de Assis D.C., Keller K.M., Campos S.V., Oliveira D.A., Fonseca L.M. (2023). Determination of the lactose content in low-lactose milk using Fourier-transform infrared spectroscopy (FTIR) and convolutional neural network. Heliyon.

[49] Min L., Fink-Gremmels J., Li D., Tong X., Tang J., Nan X., Yu Z., Chen W., Wang G. (2021). An overview of aflatoxin B1 biotransformation and aflatoxin M1 secretion in lactating dairy cows. Anim. Nutr..

[50] Jaiswal P., Jha S.N., Kaur J., Borah A., Ramya H. (2018). Detection of aflatoxin M1 in milk using spectroscopy and multivariate analyses. Food Chem..

[51] Katerinopoulou K., Kontogeorgos A., Salmas C.E., Patakas A., Ladavos A. (2020). Geographical Origin Authentication of Agri-Food Products: A Review. Foods.

[52] Stamatis C., Sarri C.A., Moutou K.A., Argyrakoulis N., Galara I., Godosopoulos V., Kolovos M., Liakou C., Stasinou V., Mamuris Z. (2015). What do we think we eat? Single tracing method across foodstuff of animal origin found in Greek market. Food Res. Int..

[53] Mendes E., Duarte N. (2021). Mid-Infrared Spectroscopy as a Valuable Tool to Tackle Food Analysis: A Literature Review on Coffee, Dairies, Honey, Olive Oil and Wine. Foods.

[54] Du L., Lu W., Gao B., Wang J., Yu L. (2019). Authenticating Raw from Reconstituted Milk Using Fourier Transform Infrared Spectroscopy and Chemometrics. J. Food Qual..

[55] Spina A.A., Ceniti C., Piras C., Tilocca B., Britti D., Morittu V.M. (2022). Mid-infrared (MIR) spectroscopy for the detection of cow’s milk in buffalo milk. J. Anim. Sci. Technol..

[56] Gonçalves B.-H., Silva G., De Jesus J., Conceição D., Santos L., Ferrão S. (2020). Fast Verification of Buffalo’s Milk Authenticity by Mid-Infrared Spectroscopy, Analytical Measurements and Multivariate Calibration. J. Braz. Chem. Soc..

[57] Souhassou S., Bassbasi M., Hirri A., Kzaiber F., Oussama A. (2018). Detection of camel milk adulteration using Fourier transformed infrared spectroscopy FT-IR coupled with chemometrics methods. Int. Food Res. J..

[58] Sen S., Dundar Z., Uncu O., Ozen B. (2021). Potential of Fourier-transform infrared spectroscopy in adulteration detection and quality assessment in buffalo and goat milks. Microchem. J..

[59] Pappas C., Tarantilis P., Moschopoulou E., Moatsou G., Kandarakis I., Polissiou M. (2008). Identification and differentiation of goat and sheep milk based on diffuse reflectance infrared Fourier transform spectroscopy (DRIFTS) using cluster analysis. Food Chem..

[60] Nicolaou N., Xu Y., Goodacre R. (2010). Fourier transform infrared spectroscopy and multivariate analysis for the detection and quantification of different milk species. J. Dairy Sci..

[61] Salleh N.A., Selamat J., Meng G.Y., Abas F., Jambari N.N., Khatib A. (2019). Fourier transform infrared spectroscopy and multivariate analysis of milk from different goat breeds. Int. J. Food Prop..

[62] Karoui R., Mazerolles G., Bosset J.-O., Debaerdemaeker J., Dufour E. (2007). Utilisation of mid-infrared spectroscopy for determination of the geographic origin of Gruyère PDO and L’Etivaz PDO Swiss cheeses. Food Chem..

[63] Woodcock T., Fagan C.C., O’Donnell C.P., Downey G. (2008). Application of Near and Mid-Infrared Spectroscopy to Determine Cheese Quality and Authenticity. Food Bioprocess Technol..

[64] Bontempo L., Barbero A., Bertoldi D., Camin F., Larcher R., Perini M., Sepulcri A., Zicarelli L., Piasentier E. (2019). Isotopic and elemental profiles of Mediterranean buffalo milk and cheese and authentication of Mozzarella di Bufala Campana PDO: An initial exploratory study. Food Chem..

[65] Caredda M., Addis M., Ibba I., Leardi R., Scintu M.F., Piredda G., Sanna G. (2016). Prediction of fatty acid content in sheep milk by Mid-Infrared spectrometry with a selection of wavelengths by Genetic Algorithms. LWT.

[66] Scampicchio M., Eisenstecken D., De Benedictis L., Capici C., Ballabio D., Mimmo T., Robatscher P., Kerschbaumer L., Oberhuber M., Kaser A. (2015). Multi-method Approach to Trace the Geographical Origin of Alpine Milk: A Case Study of Tyrol Region. Food Anal. Methods.

[67] Zaleska H., Tomasik P., Lii C.-Y. (2002). Formation of carboxymethyl cellulose–casein complexes by electrosynthesis. Food Hydrocoll..

[68] Gonçalves B.-H.R.F., Silva G.D.J., Conceição D.G., Egito A.S.D., Ferrão S.P.B. (2017). Buffalo mozzarella chemical composition and authenticity assessment by electrophoretic profiling. Semin. Ciênc. Agrar..

[69] Jiang W., Saxena A., Song B., Ward B.B., Beveridge T.J., Myneni S.C.B. (2004). Elucidation of Functional Groups on Gram-Positive and Gram-Negative Bacterial Surfaces Using Infrared Spectroscopy. Langmuir.

[70] McKnight I., Eiroa M., Sant’ana A., Massaguer P. (2010). Alicyclobacillus acidoterrestris in pasteurized exotic Brazilian fruit juices: Isolation, genotypic characterization and heat resistance. Food Microbiol..

[71] Lu X., Rasco B. (2001). Investigating Food Spoilage and Pathogenic Microorganisms by Mid-Infrared Spectroscopy. Handbook of Vibrational Spectroscopy.

[72] Quintelas C., Ferreira E.C., Lopes J.A., Sousa C. (2017). An Overview of the Evolution of Infrared Spectroscopy Applied to Bacterial Typing. Biotechnol. J..

[73] Nicolaou N., Goodacre R. (2008). Rapid and quantitative detection of the microbial spoilage in milk using Fourier transform infrared spectroscopy and chemometrics. Analyst.

[74] Moreirinha C., Trindade J., Saraiva J.A., Almeida A., Delgadillo I. (2018). MIR spectroscopy as alternative method for further confirmation of foodborne pathogens *Salmonella* spp. and *Listeria monocytogenes*. J. Food Sci. Technol..

[75] Nivens D.E., Palmer R.J., White D.C. (1995). Continuous nondestructive monitoring of microbial biofilms: A review of analytical techniques. J. Ind. Microbiol. Biotechnol..

[76] Bosch A., Serra D., Prieto C., Schmitt J., Naumann D., Yantorno O. (2005). Characterization of Bordetella pertussis growing as biofilm by chemical analysis and FT-IR spectroscopy. Appl. Microbiol. Biotechnol..

[77] Wang N., Jin Y., He G., Yuan L. (2021). Development of multi-species biofilm formed by thermophilic bacteria on stainless steel immerged in skimmed milk. Food Res. Int..

[78] Davis F., Higson S.P.J. (2010). Label-Free Immunochemistry Approach to Detect and Identity Antibiotics in Milk. Pediatr. Res..

[79] Verdini E., Pecorelli I. (2022). The Current Status of Analytical Methods Applied to the Determination of Polar Pesticides in Food of Animal Origin: A Brief Review. Foods.

[80] Casarrubias-Torres L.M., Meza-Márquez O.G., Osorio-Revilla G., Gallardo-Velazquez T. (2018). Mid-infrared spectroscopy and multivariate analysis for determination of tetracycline residues in cow’s milk. Acta Veter. Brno.

[81] Sivakesava S., Irudayaraj J. (2002). Rapid Determination of Tetracycline in Milk by FT-MIR and FT-NIR Spectroscopy. J. Dairy Sci..

[82] de Freitas A.G.M., de Magalhães B.E.A., Minho L.A.C., Leão D.J., Santos L.S., Fernandes S.A.d.A. (2020). FTIR spectroscopy with chemometrics for determination of tylosin residues in milk. J. Sci. Food Agric..

[83] de Freitas A.G.M., Minho L.A.C., de Magalhães B.E.A., dos Santos W.N.L., Santos L.S., Fernandes S.A.d.A. (2021). Infrared spectroscopy combined with random forest to determine tylosin residues in powdered milk. Food Chem..

[84] Teixeira R.C., Luiz L.C., Junqueira G.M.A., Bell M.J.V., Anjos V.C. (2020). Detection of antibiotic residues in Cow’s milk: A theoretical and experimental vibrational study. J. Mol. Struct..

[85] Soyeurt H., Bastin C., Colinet F.G., Arnould V.M.-R., Berry D.P., Wall E., Dehareng F., Nguyen H.N., Dardenne P., Schefers J. (2012). Mid-infrared prediction of lactoferrin content in bovine milk: Potential indicator of mastitis. Animal.

